# The Thinking in Motion Theoretical Model: Evaluation of Cognitive Load in Motor-Cognitive Training

**DOI:** 10.1093/geroni/igaf122.2300

**Published:** 2025-12-31

**Authors:** Shiri Embon Magal, Maayan Agmon

**Affiliations:** University of Haifa, Haifa, HaZafon, Israel; University of Haifa, Haifa, HaZafon, Israel

## Abstract

Motor-cognitive training is recognized for its role in preserving and enhancing cognitive function in aging populations. However, while sequential motor-cognitive training and dual-task paradigms are well-defined, Motor Training with Incorporated Cognitive Tasks (MTICT), where cognitive demands are integrated within movement execution, remains conceptually and practically underdeveloped, regardless of its high potential as an ecologically valid training. This gap limits research consistency, hinders comparative analysis, and reduces the ability to identify mechanisms underlying cognitive benefits of MTICT training. Furthermore, the absence of structured criteria in MTICT limits the ability to distinguish cognitively demanding interventions from general motor activities, posing challenges for both research standardization and practical application. To address these limitations, the Thinking in Motion theoretical model (TIM™), was developed. This model stems from a comprehensive review of existing theories and empirical studies examining key factors influencing cognitive load in motor learning. The TIM™ model defines five core cognitive load components: novelty, speed, variability, control complexity, and learning mode. By integrating these dimensions into standardized rating scales, TIM™ offers a structured framework for assessing both cognitive load intensity and its expected impact on cognitive domains. TIM™ refines the classification of MTICT interventions and equips clinicians and researchers with a structured tool for optimizing and evaluating cognitive stimulation in a given MTICT interventions. Standardizing cognitive load assessment in MTICT is critical for advancing both scientific methodologies and real-world applications. This presentation will outline the model’s rational, theoretical foundation, and implications for refining motor-cognitive training strategies in both clinical and community-based settings.

